# Foreign Letter

**Published:** 1890-04

**Authors:** Floyd S. Crego

**Affiliations:** Berlin


### Correspondence.

### FOREIGN LETTER.

BERLIN, February 4, 1890.

*Editors Buffalo Medical and Surgical Journal:*

As YOU doubtless have heard, I arrived safely in Berlin some days ago. That same day I took my letters of introduction to the various professors, and was very warmly received, especially by those in the nervous hospitals and clinics. I visited the clinics of my friend, Prof. Mendel, who is, perhaps, the greatest man in this specialty, (nervous

1 To relieve distension of the bladder, the catheter was used at midnight.

and mental diseases,) in Germany. I was astonished at the enormous amount of material at his polyclinic; about 300 nervous patients visit it daily from all parts of Germany, chiefly from Berlin. In every other department it is quite the same. The clinics alone run from early morning until after dark, every clinic is crowded, so that one wonders where all the sick come from. But no money or pains have been spared to make Berlin *the center* of medicine. This is generally admitted by American students to be the case. Paris, London, Vienna and New York have their especial features, but none have all departments so complete as here. This year there are about 6,000 students in Berlin, and about 2,500 of these are medical. So many lectures are going on at one time that you seldom, however, see more than 100 together, except at the anatomy lecture, and lectures of DuBois Reymond, on physiology. This morning I left my room at 8.30, and went to the Charity Hospital, where I passed through the wards devoted to nervous diseases, with Dr. Oppenheim, Chief-of-Staff of the hospital, who is a noted man in the specialty, having made many original investigations. The first case was that of a woman, about thirty years, who had left hemiplegia; at first he considered this hysteria, but upon closer investigation he found the sense of smell on the left side absent. Electrical reaction was greatly diminished, and the sense of taste somewhat impaired on the same side. This came on after a sudden attack of unconsciousness, lasting but a moment. The diagnosis was that of embolus, the exact location was somewhat doubtful on account of the complication of symptoms. A second case considered to be hysteria, was bulbar paralysis, with atrophy of the muscles of one side of the tongue; in this case a thorough electrical examination showed atrophy of the muscles of the left side with no electrical reaction but loss of sensibility, loss of sense of taste on left side of the tongue, etc. The atrophy was not at all marked when observed by the eye alone, before the application of the current. A case of disease of the cerebellum was observed, gait wide and unsteady; in turning suddenly patient lost his balance; reflexes exaggerated, instead of lost, as in tabes; dizzy most of the time. Case of spinal injury with slight curvative in lumbar region was followed by paralysis in right leg only, which was considered quite wonderful. At ten o'clock was held his polyclinic, where patients from outside are seen; those that come for the first time are seen at once and carefully examined before the class, starting with the history of the case, the examination of the head, eyes, chest, extremities electrical reactions, reflexes, etc., are all carefully gone over. A child four years of age was presented, mental faculties good, eyes normal, head symmetrical. Reflexes exaggerated, could not lift the legs, nor use the hands, so that she was wholly unable to grasp anything, unable to walk or maintain the upright position. Diagnosis, absence of cerebellum. Examination of patients continued for two hours, during which time persons with paresis, hysteria, locomotor ataxia, neurasthenia, poliomyelitis, head-aches, etc., were examined and presented. Every new patient, both in the hospital and in the clinic, who has head symptoms is examined by an oculist before a definite diagnosis is reached. One patient gave the following history: He had lived in America five years, where it was said he had epilepsy. He had been at home a week. The mother described the attacks as coming on suddenly at the dinner-table, usually, when he would throw over the table and continue for several moments to tremble and act as if frightened, or as if he saw something before him. He never lost consciousness, and when the attack was over he did not sleep. These attacks came on several times daily. Patient was hypnotized, and the suggestion made to him, while in this condition, that he would not have any more attacks. He remained in this hypnotic state about five minutes; when aroused, he was sent to the Neue Charité, a division of the hospital for epileptics, and mental diseases, where they are observed and treated, or sent to other institutions, hospital or asylum as need be, or home again. At this writing, three days later, the boy has had no more convulsions. Probably this was a case of hysteria. At eleven o'clock the clinic of Prof. Leyden took place. He presented several cases of endocarditis, which had been considered to be malarial in character. The fever chart showed exacerbations and remissions quite similar to this disease, but quinine had not affected the sense of any in the slightest. All the symptoms of endocarditis were present. He considered the course of this disease, in many cases, to be malaria, quite as often as rheumatic, and some times ill health and loss of vital power through any sickness, as pneumonia, typhus, etc., was the cause. Several cases of spinal disease, poliomyelitis, locomotor ataxia, alcoholic spine, so-called, and simple paralysis of extremities with atrophy, were presented. The first two he considered true spinal diseases, and that the first was a disease of the anterior columns of the spinal cord. That it was little understood until the discovery of the multi-polar ganglion cells in the anterior columns of the gray matter of the cord. That in poliomyelitis, otherwise known as infantile spinal paralysis, here was the first change noticed, *i. e.*, destruction of multi-polar cells: (1) Congestion of a certain portion of the cord. (2) Destruction of these cells. (3) Degeneration of the tract. (4) The sclerosis.

(5) Degeneration of nerves. (6) Muscular atrophy, etc. The symptoms are usually paralysis, after a slight fever, loss of electro-contractility of the muscles in a few days, atrophy and fatty degeneration of the muscle. He maintained that it occurred in adults less frequently than is generally supposed, such cases being mistaken in adults for simple paralysis, with atrophy, due to peripheral disease and not central. The pathological change in locomotor ataxia is well known—a sclerosis of posterior columns of the cord.

The cases of alcoholic spine he described as a neuritis, a change occurring first in the muscles, and, occasionally, the spine itself might be affected. He presented a case of paralysis of this sort, a man who, four days before, had been admitted to the hospital with complete loss of motion of the lower extremities and some loss of power of the upper extremities, loss of reflexes. No eye symptoms, as in locomotor ataxia. These symptoms had nearly all disappeared since admission, and could only be due to alcohol. Many other spinal diseases, so-called, he claimed, were previously diseases of the muscles, such as simple paralysis of muscle with atrophy paralysis, due to many poisons, such as lead, alcohol, etc.

Syphilis of the cord might be mistaken for any other of the spinal diseases, as it simulated them all, especially locomotor ataxia, or rather tabes, as it is universally called here. At twelve o'clock I went to the new charity hospital, where Dr. Siemerling, for many years the assistant of Prof. Westphal, lately deceased, gave a clinical lecture on mental diseases, lasting until 1.30 P. M. He presented several cases of paresis, one of which was totally blind; also cases of paruria. At 3 P. M. is the polyclinic of Prof. Mendel, as before mentioned. About three hundred nervous patients visit his clinic daily. To care for so many, the system must be very good. The rooms are quite perfectly arranged. The patients are received in a large waiting-room, and numbers given. Opening out of the the room are two large rooms, fitted up with all sorts of electrical apparatus. One of these is for women, one for men. In the rear of these are three rooms, one where the patients are seen who visit the institution for the first time. Again, a fifth room, for examinations, administration of oxygen, etc. In the rooms where the patients are seen for the first time are two expert assistants, who make a careful examination of each case, and note the same. Prof. Mendel visits from one room to the other, seeing each of these patients. In the other rooms, at the same time, ten patients are received at once, and treated with electricity, with suspension if the case be tabes, or new medicine ordered, as the case requires, enough assistants being present in each room to conduct the treatment properly. Dr. Rosenbaum, who has had charge of the suspension of these cases of tabes, has conducted them for over a year. The method of suspension is quite different from that first proposed by Motchoukowsky, of Odessa, and afterwards practised by Charcot. Patients are drawn quite off the floor, and from time to time are required to throw the arms gradually in the air, thus allowing the whole weight to come upon the head straps. They are also suspended longer than was at first proposed, each case being a law unto itself, as the patient bears it. Great care is also exercised in suspending patients, each case being studied carefully and observed, promiscuous suspension being sure to bring about very bad results. Dr. Rosenbaum has made over two thousand suspensions, and says the greatest improvement noticed is in the gait, Romberg's sign, bladder, symptoms, pains, etc., and that these are more or less permanent. One-fifth of all cases are benefitted by this treatment. Considering that in tabes other treatment afforded us formerly almost nothing, it is not surprising that so much is said of this form of treatment. I notice in the Berliner *Medical Weekly* several cases reported of unpleasant results in other clinics from suspension—one death, one or more followed by epileptiform convulsions. After the polyclinic proper comes an hour for the reception of patients for the treatment by massage; electricity and massage playing a very important part in the treatment of nervous diseases. At five o'clock Prof. Mendel gives a clinic on nervous and mental diseases, and once a week on nervous anatomy. Following these are lectures by Bernhardt and Remak on electro-diagnosis, and Preger on hypnotism. He believes thoroughly in the practical application of this method to diseases of the nervous system, which belief is not generally enjoyed by many here. Concerning this and other subjects I will write you. Thus is the day spent in one department alone. Even Sundays are used for visits to asylums, hospitals, etc. The influenza is about over, but many diseases, especially in the nervous line, are reported as the result of this malady.

FLOYD S. CREGO, M. D.

---

GLYCERINE SUPPOSITORIES.—Those who use Glycerine Suppositories for constipation will find that suppositories fashioned from soap, containing at least thirty per cent. of glycerine, will be a very fair substitute for the ones offered by manufacturing pharmacists, and that the chief difference to be observed is in the greater cost of the latter. —St. Louis *Weekly Medical Review*.

